# Supplementary material for: Impaired reward sensitivity in Parkinson's depression is unresponsive to dopamine treatment
Source: Brain. 2025 Mar 19;148(6):2122–34. doi: 10.1093/brain/awaf098 (PMC12129732; doi:10.1093/brain/awaf098)
Supplement: awaf098_Supplementary_Data [file awaf098_supplementary_data.pdf]

Supplementary material

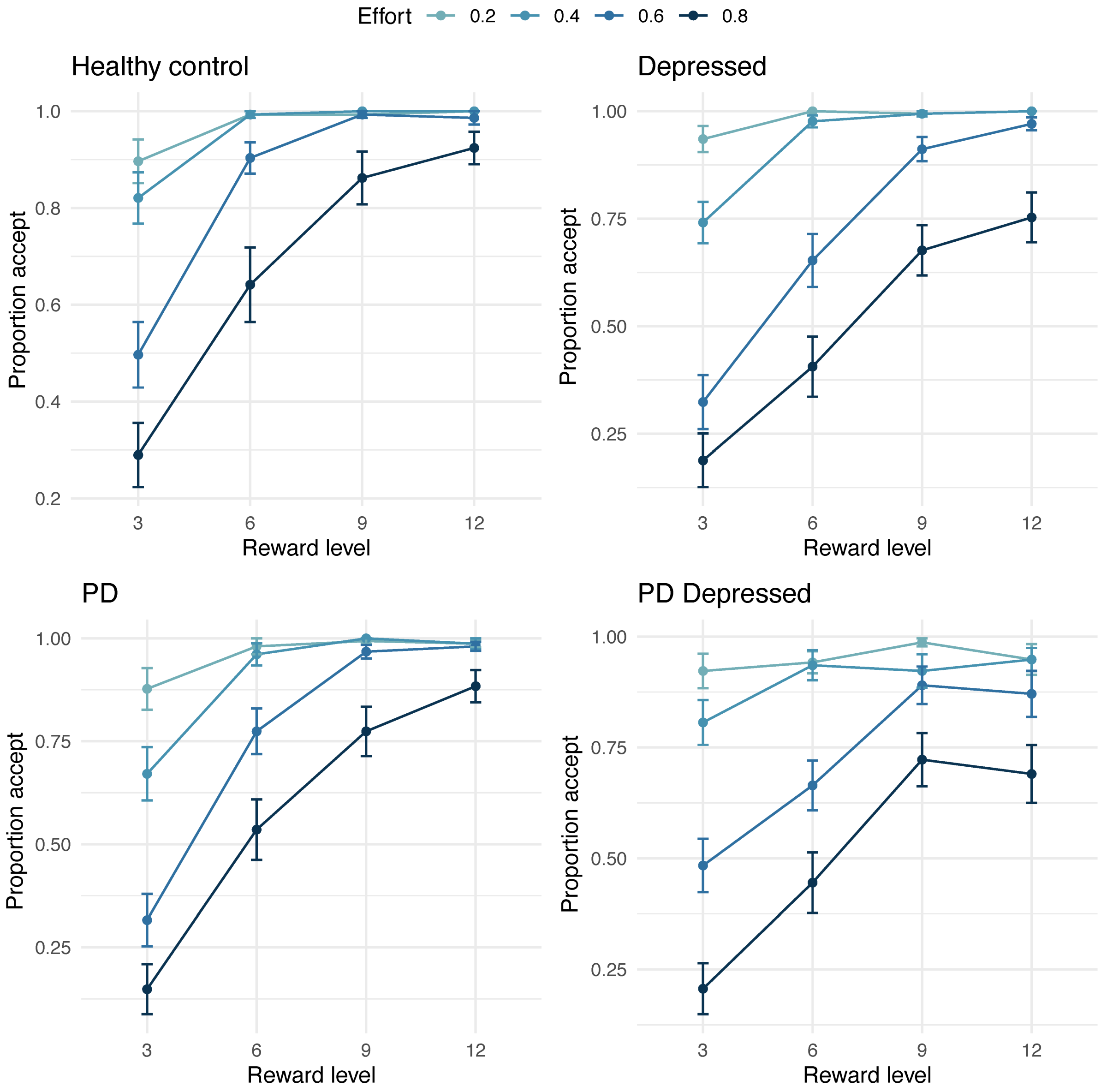

Supplemental figure S1. Empirical data showing the change in acceptance rate as reward increases for each effort level by group. (points = means, error bars = standard error)

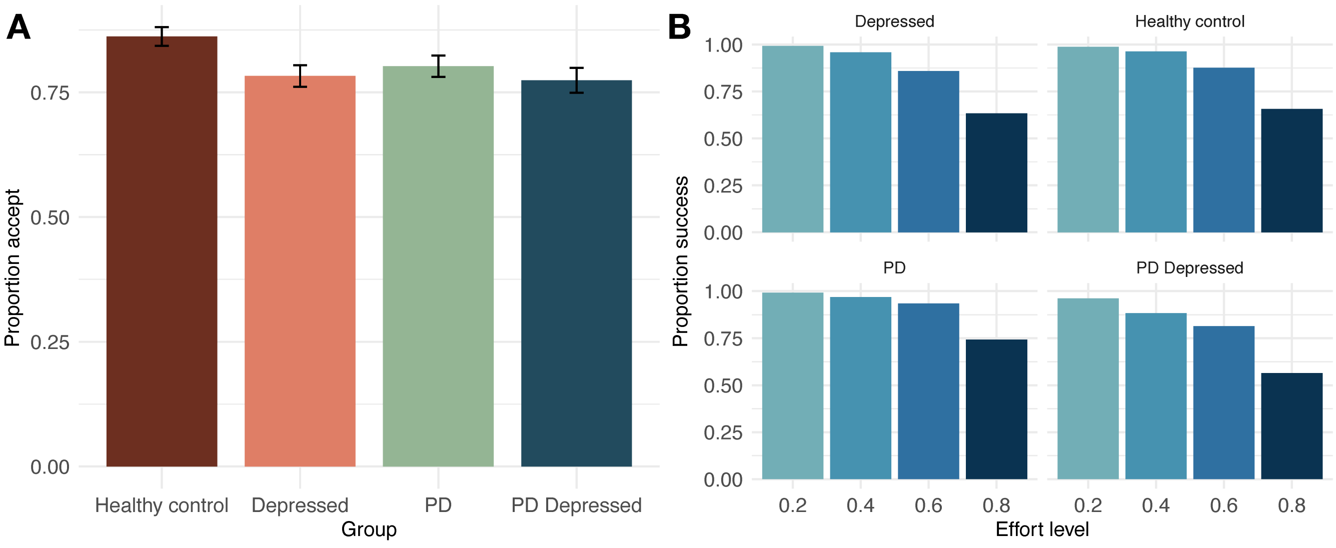

Supplemental figure S2. Empirical data showing overall accept rate by group (A) and the change in success rate as effort increases by group. (bars = means, error bars = standard error)

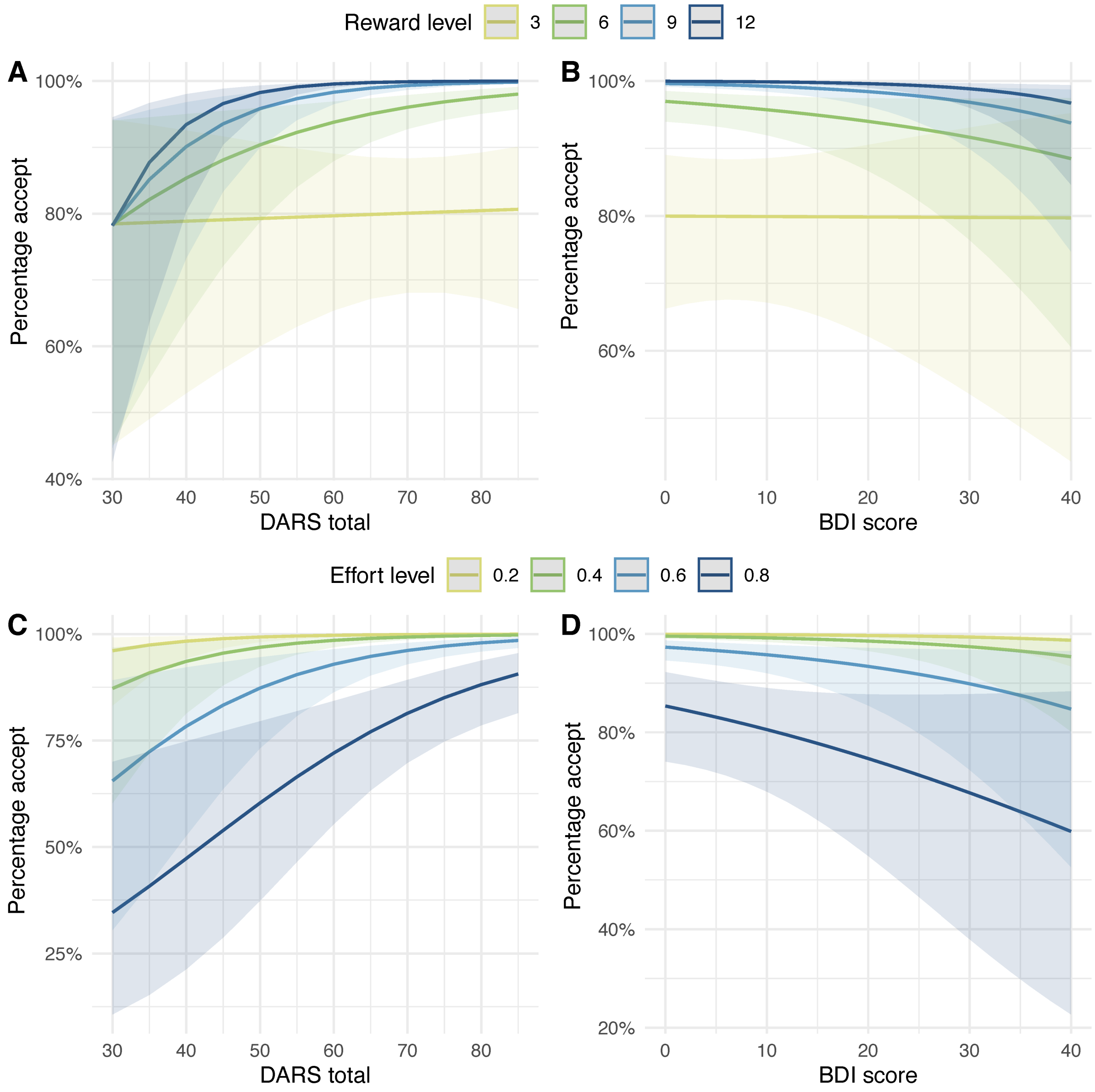

Supplemental figure S3. Model predicted plots which shows that participants who were more anhedonic (lower DARS score) or depressed (higher BDI score) were less incentivized by higher rewards to accept an offer (**A** & **B**) and more deterred by higher effort levels. (error bars = 95% confidence interval)

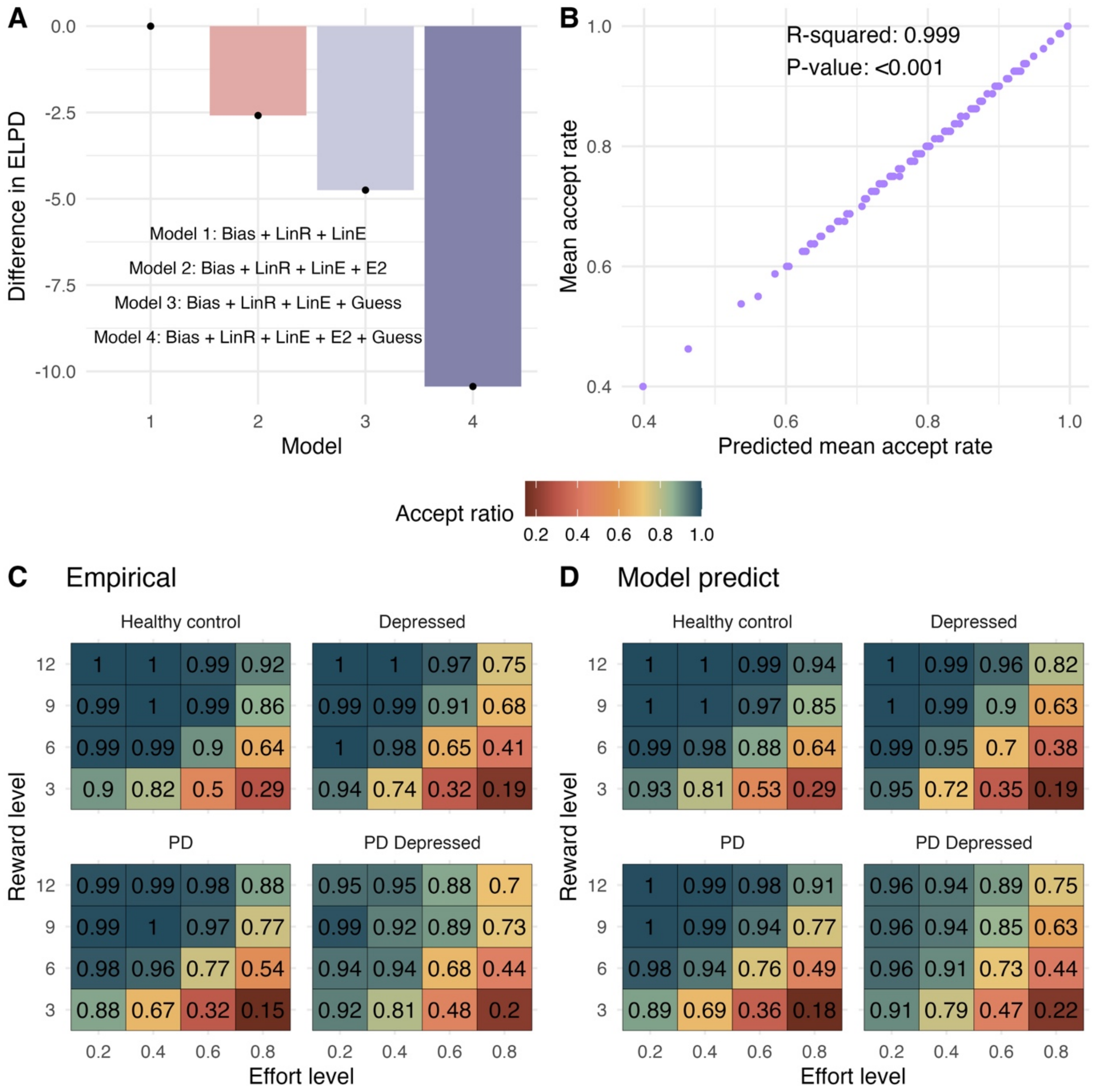

Supplemental figure S4. A. Model comparison using difference in ELPD (Expected Log Pointwise Predictive Density). ELPD measures the expected log likelihood of new data points under the model, the model with the higher ELPD is more likely to predict new data accurately. Convergence checks were conducted by visualizing trace plots and computing R-hat statistics across MCMC chains. Posterior predictive checks were conducted to ensure model predictions could accurately retrieve behavioural patterns in the original dataset.

B. Posterior predictive check plot of empirical mean individual accept rate and predicted individual mean accept rate showing almost perfect recovery of individual differences in acceptance rates. **C** & **D**. Change in each group acceptance as both reward and effort increases seen in empirical (**C**) and model predicted data (**D**), demonstrating qualitatively similar results.

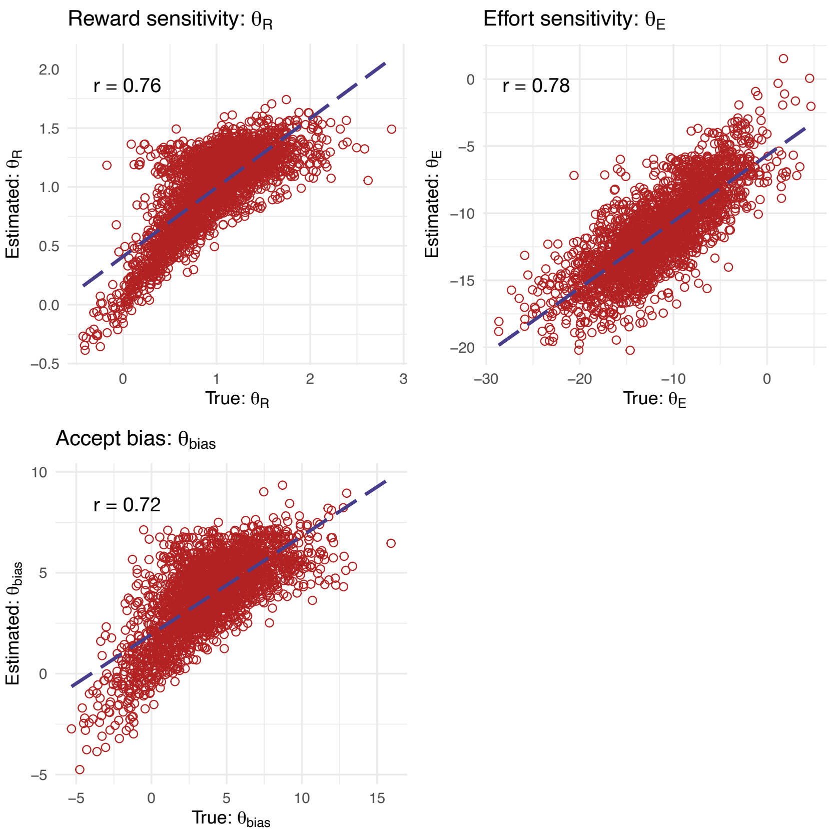

Supplemental figure S5. Parameter recovery for the winning model demonstrated excellent recovery with Pearson's r between data generating and recovered parameters ≈ 0.72 – 0.78.

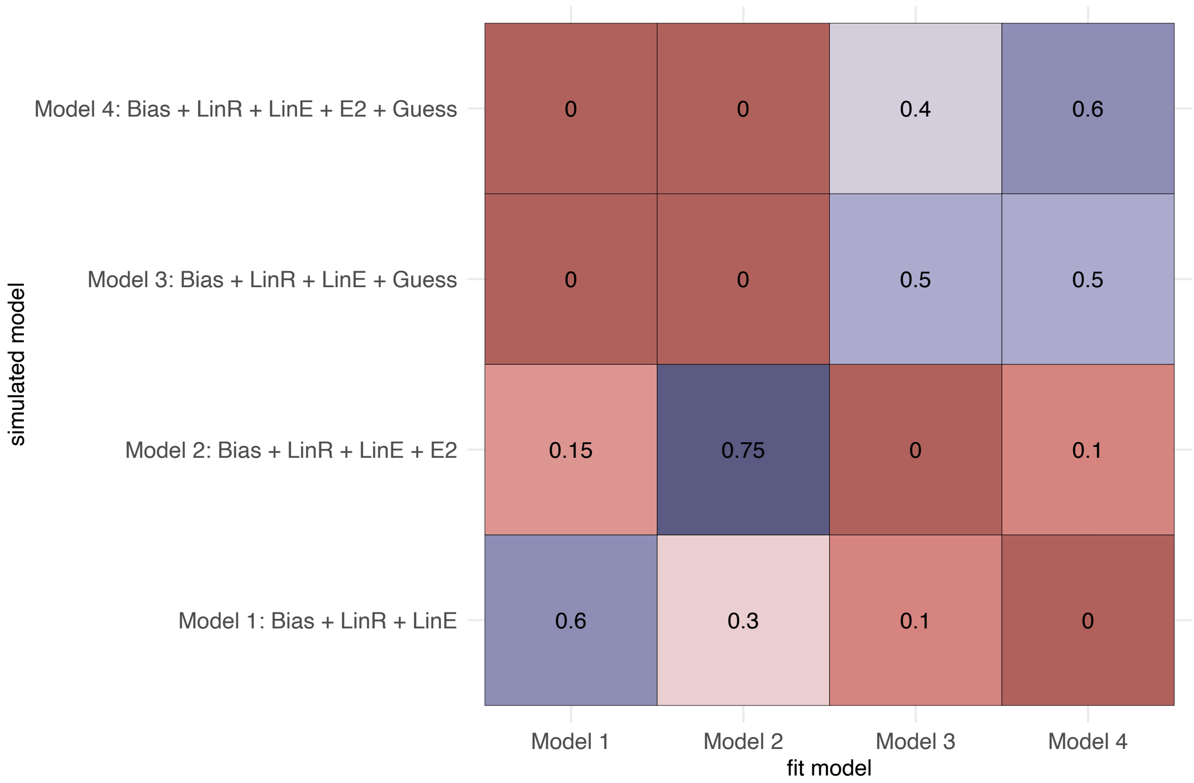

Supplemental figure S6. Confusion matrix showing the effect of prior parameter distributions on model recovery. Numbers denote the probability that data generated with model X (simulated model) are best fit by model Y (fit model).

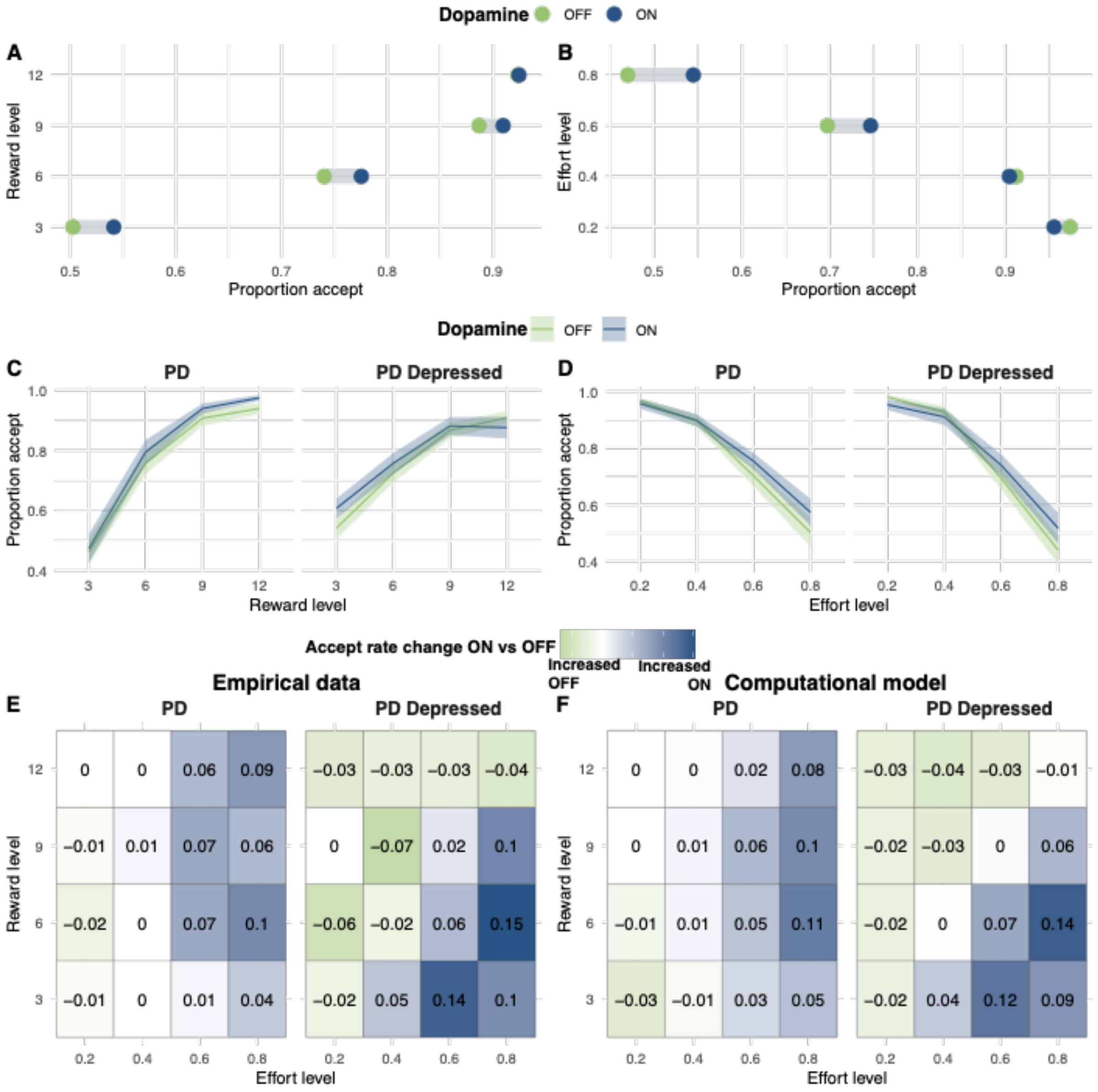

Supplemental Figure 7. Empirical data showing mean change in acceptance rate ON and OFF dopamine medication as reward (**A**) and effort (**B**) increase, across both PD groups. Acceptance rate for PD and PD depressed groups ON and OFF dopamine medication as reward (**C**) and (**D**) effort increase**.** Change in acceptance rate with dopamine medication (ON minus OFF) for PD and PD depressed groups as reward and effort increase in (**E**) empirical data and (**F**) simulated data from the Bayesian computational model.

Supplemental Table 1. Mixed effects models of group comparison with and without the addition of random effects for reward and effort to subject alone.

Group comparison Model 1: Random effects for reward, effort and subject												
**Group**	**HC**			**DEP**			**PD**			**PD+DEP**		
	**OR**	**95% CI**	**p-value**	**OR**	**95% CI**	**p-value**	**OR**	**95% CI**	**p-value**	**OR**	**95% CI**	**p-value**
**HC**		-		8.25	1.71, 39.7	0.008	3.32	0.68, 16.2	0.14	17.8	3.83, 83.0	<0.001
**DEP**	0.13	0.03, 0.61	0.01		-		0.4	0.10, 1.59	0.2	2.04	0.55, 7.51	0.3
**PD**	0.32	0.07, 1.57	0.2	2.45	0.61, 9.81	0.2		-		5.13	1.34, 19.7	0.017
**PD+DEP**	0.06	0.01, 0.29	<0.001	0.47	0.13, 1.74	0.3	0.19	0.05, 0.73	0.016		-	
**Effort*Group**	**HC**			**DEP**			**PD**			**PD+DEP**		
	**OR**	**95% CI**	**p-value**	**OR**	**95% CI**	**p-value**	**OR**	**95% CI**	**p-value**	**OR**	**95% CI**	**p-value**
**HC**		-		6.45	0.06, 722	0.4	0.51	0.00, 60.4	0.8	0.17	0.00, 17.4	0.5
**DEP**	0.12	0.00, 12.7	0.4		-		0.07	0.00, 4.59	0.2	0.03	0.00, 1.42	0.074
**PD**	1.33	0.01, 152	>0.9	11.1	0.18, 696	0.3		-		0.32	0.01, 17.4	0.6
**PD+DEP**	4.01	0.04, 393	0.6	32.8	0.64, 1,673	0.082	2.62	0.05, 141	0.6		-	
**Reward*Group**	**HC**			**DEP**			**PD**			**PD+DEP**		
	**OR**	**95% CI**	**p-value**	**OR**	**95% CI**	**p-value**	**OR**	**95% CI**	**p-value**	**OR**	**95% CI**	**p-value**
**HC**		-		1.21	0.86, 1.72	0.3	1.06	0.74, 1.51	0.8	1.84	1.30, 2.60	<0.001
**DEP**	0.83	0.59, 1.18	0.3		-		0.87	0.63, 1.18	0.4	1.49	1.11, 2.00	0.007
**PD**	0.97	0.67, 1.39	0.9	1.15	0.84, 1.57	0.4		-		1.73	1.27, 2.35	<0.001
**PD+DEP**	0.56	0.40, 0.79	<0.001	0.67	0.50, 0.89	0.006	0.58	0.43, 0.78	<0.001		-	
**Reward*Effort*Group**	**HC**			**DEP**			**PD**			**PD+DEP**		
	**OR**	**95% CI**	**p-value**	**OR**	**95% CI**	**p-value**	**OR**	**95% CI**	**p-value**	**OR**	**95% CI**	**p-value**
**HC**		-		0.94	0.44, 2.04	0.9	0.64	0.29, 1.41	0.3	0.59	0.29, 1.22	0.2

**DEP**	1.03	0.48, 2.20	>0.9	-			0.68	0.38, 1.21	0.2	0.64	0.40, 1.04	0.07
**PD**	1.48	0.68, 3.22	0.3	1.45	0.81, 2.59	0.2	-			0.93	0.56, 1.54	0.8
**PD+DEP**	1.59	0.78, 3.25	0.2	1.57	0.97, 2.54	0.067	0.06	0.64, 1.77	0.8	-		
**Group comparison Model 2: Random effects for subject alone**												
**Group**	**HC**			**DEP**			**PD**			**PD+DEP**		
	**OR**	**95% CI**	**p-value**	**OR**	**95% CI**	**p-value**	**OR**	**95% CI**	**p-value**	**OR**	**95% CI**	**p-value**
**HC**	-			5.76	1.73, 19.2	0.004	4.73	1.44, 15.5	0.01	19.5	6.25, 60.7	<0.001
**DEP**	0.18	0.06, 0.60	0.005	-			0.83	0.30, 2.25	0.7	3.44	1.35, 8.80	0.01
**PD**	0.22	0.07, 0.72	0.012	1.22	0.45, 3.34	0.7	-			4.23	1.66, 10.8	0.003
**PD+DEP**	0.05	0.02, 0.16	<0.001	0.29	0.11, 0.74	0.01	0.24	0.09, 0.61	0.003	-		
**Effort*Group**	**HC**			**DEP**			**PD**			**PD+DEP**		
	**OR**	**95% CI**	**p-value**	**OR**	**95% CI**	**p-value**	**OR**	**95% CI**	**p-value**	**OR**	**95% CI**	**p-value**
**HC**	-			0.63	0.04, 10.8	0.8	0.05	0.00, 0.79	0.034	0	0.00, 0.05	<0.001
**DEP**	1.37	0.08, 22.6	0.8	-			0.07	0.01, 0.51	0.008	0.01	0.00, 0.03	<0.001
**PD**	18.2	1.12, 295	0.041	12.9	1.89, 88.9	0.009	-			0.07	0.02, 0.34	<0.001
**PD+DEP**	246	19.4, 3,119	<0.001	174	37.2, 812	<0.001	13.2	2.89, 60.0	<0.001	-		
**Reward*Group**	**HC**			**DEP**			**PD**			**PD+DEP**		
	**OR**	**95% CI**	**p-value**	**OR**	**95% CI**	**p-value**	**OR**	**95% CI**	**p-value**	**OR**	**95% CI**	**p-value**
**HC**	-			1.28	1.06, 1.54	0.01	1.22	1.01, 1.47	0.04	1.91	1.61, 2.26	<0.001
**DEP**	0.79	0.66, 0.95	0.013	-			0.95	0.84, 1.08	0.4	1.49	1.35, 1.65	<0.001
**PD**	0.83	0.69, 1.00	0.051	1.05	0.93, 1.20	0.4	-			1.58	1.42, 1.74	<0.001
**PD+DEP**	0.53	0.45, 0.62	<0.001	0.67	0.61, 0.74	<0.001	0.64	0.57, 0.70	<0.001	-		
**Reward*Effort*Group**	**HC**			**DEP**			**PD**			**PD+DEP**		
	**OR**	**95% CI**	**p-value**	**OR**	**95% CI**	**p-value**	**OR**	**95% CI**	**p-value**	**OR**	**95% CI**	**p-value**
**HC**	-			0.55	0.28, 1.08	0.084	0.41	0.21, 0.80	0.009	0.25	0.13, 0.46	<0.001

**DEP**	1.75	0.90, 3.38	0.1	-			0.74	0.46, 1.19	0.2	0.44	0.30, 0.65	<0.001
**PD**	2.36	1.21, 4.60	0.012	1.35	0.83, 2.17	0.2	-			0.59	0.40, 0.89	0.011
**PD+DEP**	3.96	2.16, 7.27	<0.001	2.26	1.53, 3.33	<0.001	1.67	1.12, 2.49	0.012	-		
